# Bladder Mucosa Harvested with Holmium Laser for Treatment of Urethral Strictures: Does the Graft Have its Tissue Integrity Preserved?

**DOI:** 10.1590/S1677-5538.IBJU.2024.9923

**Published:** 2025-01-03

**Authors:** Luiz Augusto Westin, Edilaine Farias Alves, Waldemar S. Costa, Francisco J. B. Sampaio, Luciano A. Favorito

**Affiliations:** 1 Universidade do Estado do Rio de Janeiro Unidade de Pesquisa Urogenital Departamento de Urologia Rio de Janeiro RJ Brasil Unidade de Pesquisa Urogenital, Departamento de Urologia - Universidade do Estado do Rio de Janeiro, UERJ, Rio de Janeiro, RJ, Brasil

**Keywords:** Urethral Stricture, Mucous Membrane, Histology

## Abstract

**Objective:**

The aim of this study is to evaluate the integrity and the microstructural characteristics of the bladder mucosa graft harvested using a minimally invasive technique with the Holmium laser (Ho-YAG) for the treatment of urethral stricture.

**Materials and Methods:**

We studied patients with urethral strictures greater than 2 cm, with a urethroplasty indication. The patients were submitted to urethroplasty with the dorsal onlay reconstruction by a single surgeon. After the urethral dissection we use the Ho-YAG laser with a 550µg end fire laser fiber to obtain a fragment of bladder mucosa for the graft confection. A fragment of the bladder mucosa was fixed in a 10% buffered formalin to HE and Masson's trichrome analysis for the tissue integrity. Five sections were stained, and five fields of each section were selected. We used the Image J software, version 1.46r, loaded with its own plug-in to determine tissue integrity.

**Results:**

We studied 11 patients (Mean age= 47.64); 9 (81.8%) with bulbar stricture and 2 (18.2%) with penile stricture (mean size = 4.63mm). The mean of bladder graft size was 53.64mm and the meantime of harvesting was 47.63 minutes. The histological study of the bladder wall graft showed an organization in accordance with normal standards, with the presence of an intact urothelium in the bladder graft. The submucosal layer is preserved, joining the detrusor to the urothelium and the collagen and elastic fibers are well organized.

**Conclusion:**

The graft harvested from the bladder uroepithelium using Ho-YAG has its histological integrity preserved, which makes this technique a viable option for reconstructive surgery. However, more studies are needed to establish its long-term efficacy and safety of this new technique.

## INTRODUCTION

The use of oral mucosa as a graft for the treatment of urethral stricture is well established, but not free from morbidity (1). Bladder mucosa has been utilized in various forms of urethral reconstruction, particularly in cases where other graft materials are not suitable or available (2, 3). For instance, Ozgök et al. demonstrated the use of bladder mucosa grafts in urethral reconstruction for patients with penoscrotal or scrotal hypospadias, showing a complication rate of 28.6% (4). Similarly, Monfort et al. reported successful outcomes using bladder mucosa grafts for urethral strictures in children, with most patients achieving satisfactory results (5). Additionally, Garat and Villavicencio described the use of tubularized bladder mucosal grafts for posterior urethroplasty, indicating good initial results in challenging cases (6).

More recent techniques, such as those described by Westin et al., involve the use of Holmium: YAG laser for transurethral harvesting of bladder mucosa, which has shown promising preliminary results for dorsal onlay urethroplasty (7). These studies collectively support the feasibility and effectiveness of bladder mucosa as a graft material in urethroplasty, particularly in complex or recurrent cases where other graft options may be limited.

Studies showing the histological integrity of bladder mucosa graft removed using laser have never been done. Our hypothesis is that laser removal of the bladder mucosa preserves the tissue integrity of the graft. The aim of this study is to evaluate the integrity and the microstructural characteristics of the bladder mucosa graft harvested using a minimally invasive technique with the Holmium laser (Ho-YAG) for the treatment of urethral stricture.

## MATERIALS AND METHODS

The study was approved according to the ethical standards of the hospital's institutional committee on experimentation with human beings (IRB number 51456521.8.0000.5259).

We prospectively analyzed 11 patients, admitted to our service between November 2021 and January 2024. Inclusion criteria consisted of patients having a diagnosis of anterior urethral stenosis, with or without recurrence, and were indicated for urethroplasty with graft (strictures greater than 2.5 cm). Exclusion criteria included: genitourinary malformations, a history of pelvic radiotherapy, a history of bladder cancer, and those with an indication for staged urethroplasty. Every patient was staged using cystourethrography and uroflowmetry except in those using a suprapubic urinary diversion.

All surgeries were performed by a single surgeon with experience in urethral surgery. Due to the physical characteristics of the bladder mucosa (soft and tenacious tissue), we chose to perform dorsal onlay (8) or dorsum lateral onlay urethroplasty (9) to avoid diverticula formation. After placing the patient in the lithotomy position, a perineal incision was made permitting access to the bulbar urethra. The next step proceeded with either the dorsal or lateral dorsum urethral dissection and following with location of the stenosis aided by a urethral catheter, longitudinal section, and measurement of the strictured urethral segment until reaching the suspected healthy proximal and distal urethral areas.

A 22 or 18.5F resectoscope with a working element adapted for the laser fiber was then passed through the proximal urethrostomy followed by a urethroscopy and cystoscopy using a 0.9% saline solution as irrigation fluid (10). This is performed to aid in identifying possible bladder and/or urethral pathologies and anatomical landmarks for marking the graft donor region. The Holmium Laser settings for energy were 0.5 to 0.8J and frequency of 30 to 40 Hz. After filling the bladder to full capacity, a rectangular marking of the donor graft area was made immediately above the inter-ureteral bar (10).

Dissection of the graft was then performed using the 550μm laser fiber, always going from lateral to medial and subsequently from proximal to distal, being that the deepest plane is the muscular layer of the bladder. Upon completing dissection, the graft is extracted from the bladder's interior using forceps and hemostasis then performed on the edges of the donor area and a small fragment of the graft was removed for histological analysis.

The fragment of bladder mucosa was fixed in 10% buffered formalin, and routinely processed for paraffin embedding, after which 5µm thick sections were obtained at 15 µm intervals and studied by histochemical methods. The sections were stained with hematoxylin-eosin to assess the integrity of the tissue. We also performed the staining with Masson's trichrome Five sections were stained, and five fields of each section were selected. All selected fields were photographed with a digital camera (Olympus DP70, Tokyo, Japan) under the same conditions at a resolution of 2040 × 1536 pixels, directly coupled to the microscope (Olympus BX51, Tokyo, Japan) and stored in a TIFF file. We used the Image J software, version 1.46r, loaded with its own plug-in to determine tissue integrity.

## RESULTS

We can observe the demographic data and the etiology of urethral strictures of the patients studied in Table-1. The patients’ ages ranged from 31 to 70 years old (mean= 53.45). The mean of bladder graft size was 53.64mm (4 to 7 cm) and the meantime of harvesting was 47.63 minutes (75 to 25 minutes).

**TABLE 1:** The table shows the demographic data of the 11 patients submitted to urethroplasty with bladder mucosa graft.

Patient	Age	Comorbidities	Etiology	Prior Urethral Manipulation	Preoperative urethrocystogroma	Operative technique	Graft harvest duration	Grafit Size
1	39	No	Idiopathic	No	Bulbar urethral stricture 5 cm	Kulkarni	60 min	6 cm
2	48	SAH	Straddle Injury	No	Bulbar urethral stricture 3 cm	Augmented Anastomotic Urethroplasty	25 min	5 cm
3	63	Morbid obessity and SAH	Pelvic Trauma	No	Bulbar urethral stricture 4 cm	Kulkarni	30min	6 cm
4	53	No	Pelvic Trauma	Urethroplasty	Bulbar Stop	Augmented Anastomotic Urethroplasty	45 min	4 cm
5	45	No	Idiopathic	No	Bulbar urethral stricture 3 cm	Kulkarni	45 min	7 cm
6	46	No	Pelvic trauma	Urethroplasty	Bulbar urethral stricture 3,5 cm	Kulkarni	60min	5 cm
7	31	Morbid obesity and SAH	Idiopathic	No	Bulbar urethral stricture 4 cm	Kulkarni	75 min	6 cm
8	62	SAH	Idiopathic	No	Bulbar urethral stricture 3 cm	Kulkarni	35 min	5 cm
9	62	SAH	Indwelling Urinary Catheter	No	Bulbar urethral stricture 3 cm	Kulkarni	30min	6 cm
10	49	No	Idiopathic	DVRU	Bulbar urethral stricture 4 cm	Kulkarni	25 min	6 cm
11	70	SAH and Diabetes	Indwelling Urinary Catheter	No	Bulbar urethral stricture 3,5 cm	Kulkarni	32min	4 cm

**Figure 1:**
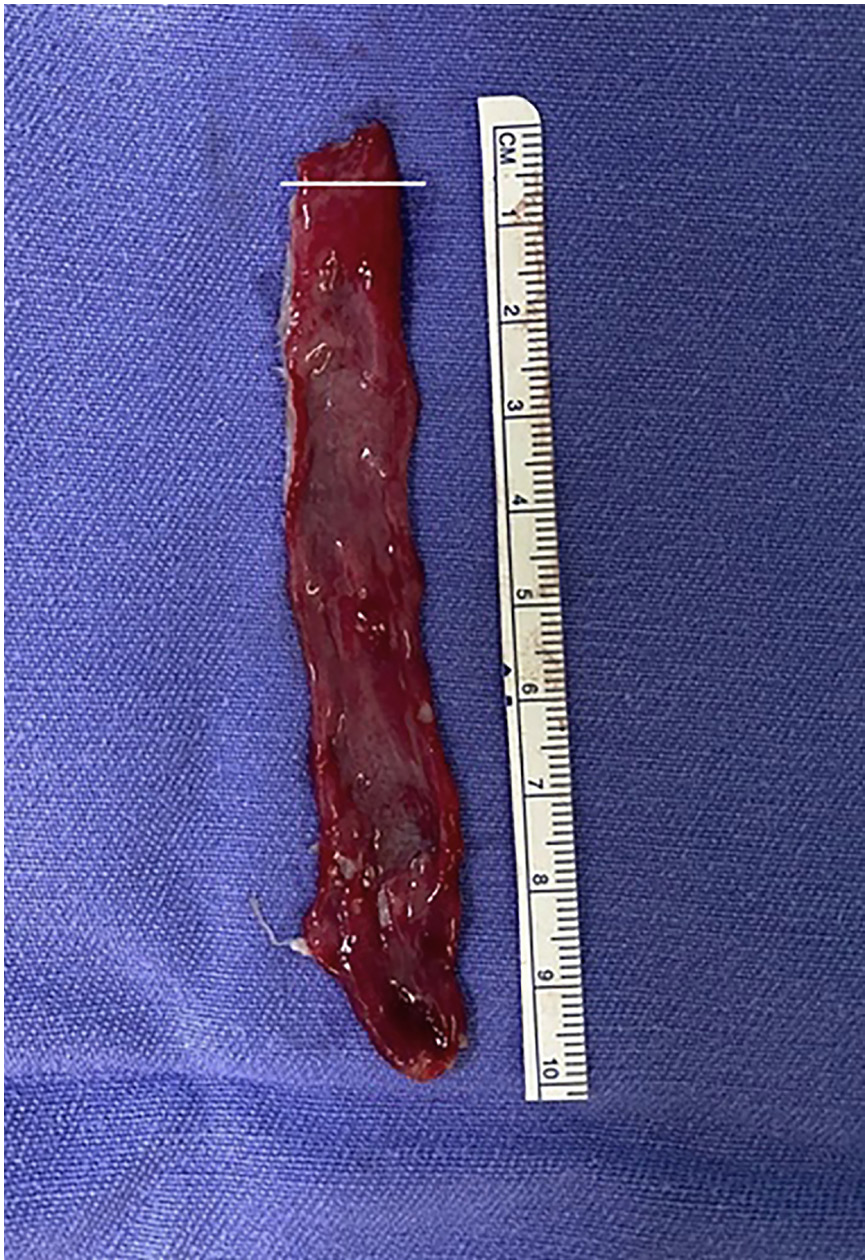
The figure shows the final aspect of bladder mucosa graft harvested with Holmium laser. The white line shows the fragment that was used to histological analysis.

**Figure 2:**
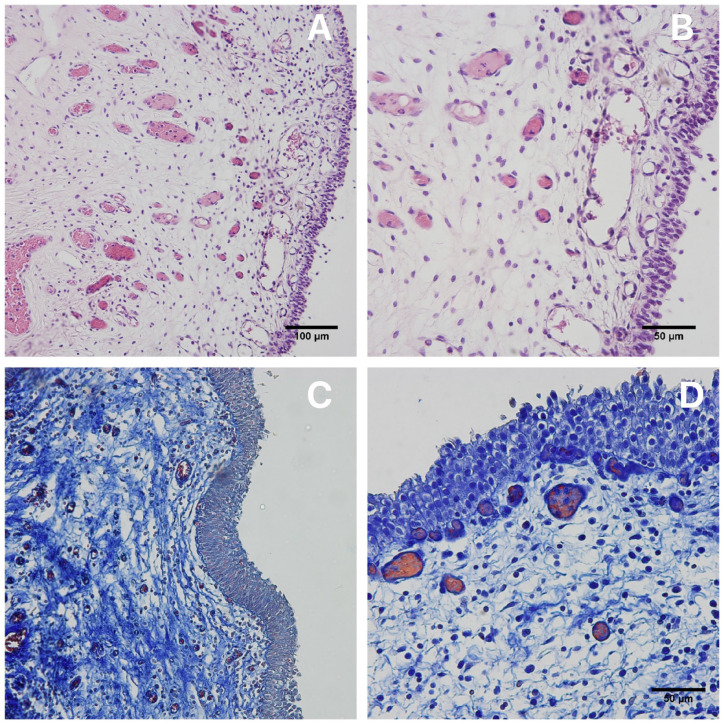
A) Photomicrograph showing the integrity of the bladder mucosa graft harvested with Holmium laser. HE X20 Masson's trichrome X1000; B) Photomicrograph showing of the bladder mucosa graft in higher augmentation. HE X40. C) Photomicrography showing the integrity of bladder mucosa. Masson's trichrome X20; and D) Photomicrography showing the bladder epithelium and mucosa structure of the graft harvested with Holmium laser Masson's trichrome X20.

The histological study of the bladder wall graft showed an organization in accordance with normal standards, with the presence of an intact urothelium in the bladder graft with no signs of compromise after laser removal (Figure-1). The bladder mucosa graft was lined by transitional epithelium (urothelium), which is composed of multiple layers of cells. The submucosal layer was preserved, joining the detrusor to the urothelium and the collagen and elastic fibers were well organized. The lamina propria lies beneath the urothelium and is composed of loose connective tissue containing blood vessels, nerves, and lymphatics and contains wispy, slender fascicles of the muscularis mucosae (MM), which can appear as individual or small groups of wavy muscle fibers (Figure-2).

## DISCUSSION

The use of a laser to collect bladder mucosa for urethroplasty is possible and was described for the first time by Joseph Memmelaar, in 1947 (11) for the treatment of 4 patients with hypospadias. Applying the knowledge and technology of that time, the grafts were harvested using an open technique and tubularized for the repair of hypospadias in a 1-stage procedure, obtaining patency in 3 out of 4 patients after 1 year.

Specifically, the Holmium: YAG (Ho:YAG) laser has been utilized for this purpose. A recent study shows a technique for transurethral harvesting of bladder mucosal grafts using the Ho:YAG laser. This technique was applied in a series of patients undergoing dorsal onlay urethroplasty for anterior urethral stricture. The results indicated that the procedure is feasible and reproducible, with comparable outcomes to other graft types used in urethroplasty (7). Another study by Figueiredo et al. also supports the feasibility of using the Ho:YAG laser for endoscopic harvesting of bladder mucosal grafts (10). This study described the successful application of this technique in a patient with a bulbar urethral stricture, further suggesting that bladder mucosal grafts harvested with the Ho:YAG laser could be a viable alternative to buccal mucosa grafts in urethral reconstruction (10).

The use of buccal mucosa for urethroplasty has been shown to retain its histopathological characteristics after engraftment to the urethra. Soave et al. found that buccal mucosa transplants maintain their structure and are not overgrown with urothelium after being integrated into the urethra (12). In our study we observed the preservation of the histology of bladder mucosa during the resection of graft with laser. According to a study by Li et al., the freeze-thaw technique can maintain the structure and biological function of bladder mucosa. The study demonstrated that no significant histological changes were observed in the frozen-thawed bladder mucosa compared to fresh bladder mucosa, and the urethral epithelial cells grew well postoperatively (13).

In our paper we studied the bladder histology with hematoxylin and eosin (H&E) and Masson's trichrome. The bladder mucosa, when stained with H&E, exhibits several distinct histological characteristics characterized by the transitional epithelium, a supportive lamina propria with variable muscle fiber patterns, and a deeper muscularis propria with more organized muscle bundles (14), which gives us support for the structural analysis of bladder mucosa in our study. In the study by Julio Junior et al., Masson's trichrome stain was used to quantify connective tissue and smooth muscle in the bladder structure of fetuses with Prune Belly syndrome (15). This demonstrates the utility of Masson's trichrome stain in analyzing the structural components of the bladder mucosa. Additionally, Paner et al. utilized Masson's trichrome stain to differentiate between muscularis propria and muscularis mucosae in the urinary bladder, further supporting its application in detailed structural analysis of bladder tissues (16). Thus, Masson's trichrome stain is a valuable tool for examining the structural details of the bladder mucosa, particularly in distinguishing between different tissue types such as collagen and smooth muscle.

The present paper has some limitations: small sample, lack of ultra-structural analysis of bladder mucosa and longer follow-up.

In conclusion, our findings suggests that the graft harvested from the bladder uroepithelium using Ho-YAG has its histological integrity preserved, which makes this technique a viable option for reconstructive surgery. However more studies are needed to establish its long-term efficacy and safety of this new technique.
